# Who has the responsibility to inform relatives at risk of hereditary cancer? A population-based survey in Sweden

**DOI:** 10.1136/bmjopen-2024-089237

**Published:** 2024-11-27

**Authors:** Kalle Grill, Amicia Phillips, Barbro Numan Hellquist, Anna Rosén

**Affiliations:** 1 Historical, Philosophical and Religious studies, Umeå University, Umeå, Sweden; 2 Centre for Biomedical Ethics and Law, Department of Public Health and Primary Care, KU Leuven, Leuven, Flanders, Belgium; 3 Department of Clinical and Biomedical Sciences, University of Exeter, Exeter, UK; 4 Diagnostics and Intervention, Oncology, Umeå University, Umeå, Sweden

**Keywords:** Cancer genetics, GENETICS, Risk management, MEDICAL ETHICS, Gastrointestinal tumours

## Abstract

**Abstract:**

**Objectives:**

Hereditary cancer has implications not only for patients but also for their at-risk relatives (ARRs). In current clinical practice, risk disclosure to ARRs involves collaboration between patients and healthcare providers (HCPs). However, the specific responsibilities of each party are intertwined and at times unclear. In this study, we explored public attitudes regarding moral and legal responsibilities to disclose familial risk information to uninformed ARRs.

**Design:**

In an online cross-sectional survey, participants were prompted with a hypothetical scenario where a gender-neutral patient learnt about their familial risk of colorectal cancer. The patient was advised to regularly undergo colonoscopy screening, and this recommendation was extended to both their siblings and cousins. While the patient informed their siblings, they had not spoken to their cousins in 20 years and did not want to contact them. The survey assessed respondents’ views on the patient’s and HCPs’ ethical responsibility and legal obligation to inform the cousins (ARRs).

**Participants:**

A random selection of 1800 Swedish citizens 18–74 years of age were invited. Out of those, 914 (51%) completed the questionnaire.

**Results:**

In total, 75% believed that HCPs had a moral responsibility to inform ARRs, while 59% ascribed this moral responsibility to the patient. When asked about the ultimate responsibility for risk disclosure to ARRs, 71% placed this responsibility with HCPs. Additionally, 66% believed that HCPs should have a legal obligation to inform ARRs, while only 21% thought the patient should have such an obligation. When prompted about a scenario in which the patient actively opposed risk disclosure, a majority believed that HCPs should still inform the ARRs.

**Conclusion:**

Our study indicates that the Swedish public ascribes moral responsibility for informing ARRs to both the patient and HCPs. However, contrary to current practice, they believe HCPs hold the ultimate responsibility. The majority of respondents support disclosure even without patient consent.

**STRENGTHS AND LIMITATIONS OF THIS STUDY**

- The invited sample (n=1800) was stratified to gain a study population being representative of the Swedish general population between 18 and 74 years of age.
- The response rate was relatively high for a population-based survey (51%).
- The generalisability of our findings is limited by an over-representation of respondents at a higher age, with higher education, and those born in Sweden, as well as by the fact that our data was collected in 2018.
- The data set allows for subset analysis by sex, age, educational level, country of birth, having children, cancer history and preferences regarding hereditary cancer risk disclosure.
- We acknowledge that the reported participant attitudes are based on hypothetical scenarios, which may differ from perspectives informed by real-life experiences.

## Introduction

 Identifying families with a confirmed familial risk or high-risk genetic variant associated with a predisposition for colorectal cancer is an important strategy for targeted cancer prevention, given that surveillance of at-risk relatives (ARRs) reduces both cancer incidence and mortality.^1^,^3^ However, the effectiveness of targeted prevention in high-risk families depends on the uptake of testing and surveillance in ARRs.^4^

One crucial factor affecting the uptake of genetic counselling and testing is the dissemination of correct information to ARRs. Such dissemination involves several steps or dimensions. ARRs must be identified, their contact data must be obtained and they must be effectively reached by some means of communication. Once ARRs have information at hand, it must be accurate and they must understand it. Several patient-related and interpersonal factors have been identified as barriers (and facilitators) in the communication chain from the first counselling of the index patient to ARRs approaching the clinic.^5 6^ Interventions attempting to overcome the barriers and improve the support provided by healthcare providers (HCPs) have not been very effective.^7^ One overarching factor that could help determine how these various dimensions are best addressed is clarity around who is responsible for informing ARRs.

With a few exceptions, in Europe, the current information dissemination paradigm is that while HCPs should support the index patient in informing ARRs, the ultimate responsibility for doing so belongs to the index patient.^8^ This paradigm influences clinical practice, as evidenced by a reliance on the so-called ‘family-mediated disclosure’ to ARRs. Ethically speaking, however, this paradigm is controversial.^9^,^12^ Patients may have a moral duty to inform their ARRs, but it is not clear what mandate HCPs have to induce or pressure them to conform to that duty.^13 14^ When effective treatment is available for ARRs, informing them is a means of health promotion, but it is not clear how this general goal should inform the responsibility of individual HCPs.

The duty to maintain confidentiality that HCPs owe patients, the ARRs’ (potential) right not to know and the practical challenges involved in finding and informing ARRs, could mean that it is not within HCPs’ professional responsibility to inform.^10 14^ On the other hand, HCPs as a collective could have such a responsibility, even if it is constrained by or coexists with other duties, based on their opportunity and ability to inform, in combination with a general duty to promote and protect population health, as well as a duty to empower individuals to protect their own health.^13^

This background of ethical uncertainty makes it particularly worthwhile to investigate public opinion regarding these contentious issues around the disclosure of genetic information to ARRs. Not because this will decide the ethical matter, but because it may provide information on widespread moral sentiments and expectations that HCPs—and the healthcare authorities—need to accommodate in one-way or another, either by aligning with them or by constructively opposing them and providing arguments for an alternative approach.

In this article, we investigate public attitudes toward patients’ and HCPs’ moral responsibility for risk disclosure to ARRs in Sweden. We also report what the Swedish public think about patients’ and HCPs’ legal obligations to inform ARRs and how they think HCPs should handle a situation where a patient explicitly says they do not want to inform ARRs.

## Method

### Context: Swedish healthcare

The Swedish healthcare system is decentralised and managed by regional authorities. The entire Swedish population has equal access to healthcare according to the Health and Medical Service Act. The public’s level of trust in HCPs is fairly high compared with citizens in other European countries.^15 16^ Investigations for hereditary cancer predisposition syndromes are offered at public specialised clinics in seven university hospitals nationwide. If an individual needs treatment or surveillance (like colonoscopy), the patient fee and travel to care is subsidised by taxes, with high-cost protection.

The Swedish national legislation does not address genetic counselling.^17^ However, in the preparatory works to the Genetic Integrity Act (2006:351), it is noted that HCPs may inform ARRs directly about the results of a genetic test if the patient consents. Circumstances in each case should guide whether the disclosure to ARRs should be handled by the patient or by HCPs.

### Patient and public involvement

The questionnaire was developed by the research group based on insights from prior qualitative content analysis of explorative patients interviews^18^ and focus group discussions with the public.^19^ Patients and the public were not involved in the conduct, reporting or dissemination plans of this research.

### Data collection and analysis

Participants were recruited through the digital research infrastructure Citizen Panel, hosted by the Laboratory of Opinion Research (LORE) at the University of Gothenburg, Sweden.^20^ We invited a stratified sample of panellists that had previously been recruited to the Citizen Panel from a randomly selected sample of the Swedish Population Register Survey data by distributing an electronic questionnaire.^21^ Data were collected between the 12 September and the 7 October 2018. Two electronic reminders were sent to non-responders after the initial survey distribution. Self-reported information about participants’ sex, age, education level, country of birth and having children were acquired from the Citizen Panel.

Respondents received a general introduction to hereditary cancer care, after which they were presented with six different scenarios. The first four scenarios concerned attitudes towards hereditary cancer risk information.^22^ In this article we report on the fifth scenario, henceforth referred to as ‘the scenario’. In the scenario, a gender-neutral person named Kim, aged 40, undergoes an investigation concerning hereditary cancer and is informed by HCPs that the results concern both Kim and their ARRs (box 1).

Box 1 **The scenario setting the scene for a cancer genetic investigation with implications both for the patient and their at-risk relatives.**

Kim, 40 years old, has initiated a cancer genetic investigation because several of Kim’s relatives had colorectal cancer rather young. The investigation shows that Kim, Kim’s siblings and Kim’s cousins may have an increased risk of developing colorectal cancer. They can be offered regular colonoscopies. Kim informs the siblings but has not spoken with the cousins for 20 years and does not want to contact them.

The questionnaire explored the respondents’ attitudes towards moral and legal responsibility to inform ARRs through questions with four Likert scale response alternatives in rank order. The respondents were also asked which party they considered ultimately responsible for informing the ARRs (with response alternatives the index patient, HCPs or others). The scenario develops into a situation where Kim objects to disclosing information to the cousins, and respondents were asked if they thought HCPs should inform the cousins against Kim’s will.

Participants’ attitudes on moral and legal responsibility are described and analysed in subgroups according to sex, age, educational level, country of birth, having children, cancer history and their preferences on risk disclosure. We also relate respondents’ attitudes in this scenario with their preferences from previous scenarios on whether they want to be informed about a potential hereditary risk for developing colorectal cancer, and whether they want their relatives to be informed about such a risk (lifetime risk of 10% instead of population risk of 5%). The questionnaire was administrated in Swedish (online supplemental table 6). Translation of the scenario and follow-up questions, and response rate for all items, can be found in the supplementary information (online supplemental table 1).

### Statistical methods

Categorical variables are described with counts and proportions and compared using χ^2^ tests. A p value below 0.05 was considered statistically significant. The statistical software package R, V.3.5.2 was used for data analysis and creation of figures.^23^

## Results

### Study population

Of the 1800 invited, 977 responded. Only those who had responded to all questions in the scenario were included in the study population (n=914). Respondents of a higher age, with high levels of education and born in Sweden were over-represented compared with the general Swedish population (table 1).

**Table 1:** Characteristics of the Swedish population and respondents

	Subgroup	Population Sweden*		Respondents		Χ^2^ test
		N	%	N	%	
Total	–	7 152 054	–	914	–	
Gender	Men	3 633 651	51	481	53	
	Women	3 518 403	49	433	47	0.29
	NA	0	0	0	0	
Age	18–29	1 562 778	22	123	13	
	30–39	1 330 260	19	137	15	
	40–49	1 294 175	18	157	17	
	50–59	1 286 816	18	150	16	
	60–69	1 114 377	16	193	21	
	70–74	563 648	8	154	17	<0.0001
	NA	0	0	0		
Education†	Lower	4 219 613	59	366	40	
	Middle	1 072 193	15	291	32	
	Higher	1 680 357	23	252	28	<0.0001
	NA	179 891	3	5	1	
Country of birth‡	Sweden	5 537 132	77	843	92	
	Other	1 614 922	23	63	7	<0.0001
	NA	0	0	8	1	
Children§	Yes	4 577 315	64	598	65	
	No	2 574 739	36	311	34	0.28
	NA	0	0	5	1	

* Swedish population data on number of individuals aged 18–74 years in 2018 retrieved from officially available reports by Statistics Sweden ().

† Lower - —elementary or high school education, Mmiddle - —post-secondary education <3 years, or Hhigh - —3 years of post-secondary education or more.

‡ Self-reported country of birth with response options; Sweden, Europe, or Ooutside Europe.

§ Respondents’ answers to the question; ‘“Do you have children?’.

### Moral responsibility to inform ARRs?

**Figure 1:**
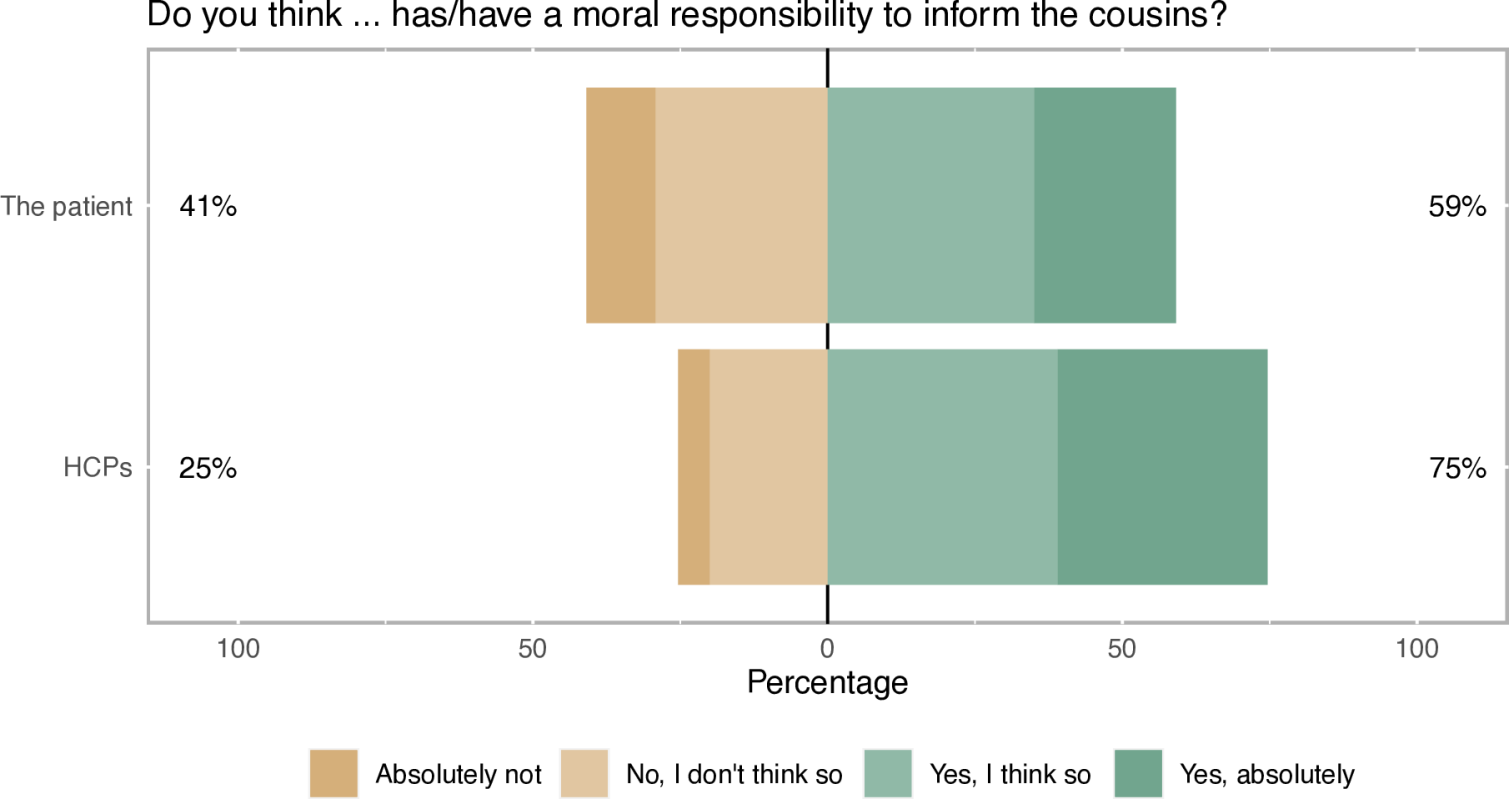
Public attitudes on the patient’s and healthcare providers’ (HCPs’) moral responsibility to inform at-risk relatives.

In univariable analysis, 59% ascribed a moral responsibility to the patient and 75% to HCPs (figure 1). Cross-tabulation of these questions showed that 51% of respondents held both the patient and HCPs responsible, while 24% thought only HCPs had a moral responsibility and 8% thought only the patient had a moral responsibility (online supplemental figure S1). A larger proportion of young respondents ascribed a moral responsibility to HCPs as compared with older respondents (p<0.001). Among those who would like to be informed about a potential risk for colorectal cancer, and those who wanted their relatives to be informed about such risk, a significantly larger proportion ascribed a moral responsibility to the patient, as well as to HCPs, compared with those who did not want to be informed, or did not want their relatives to be informed (online supplemental table 2).

### Who should have the ultimate responsibility for informing ARRs?

**Figure 2:**
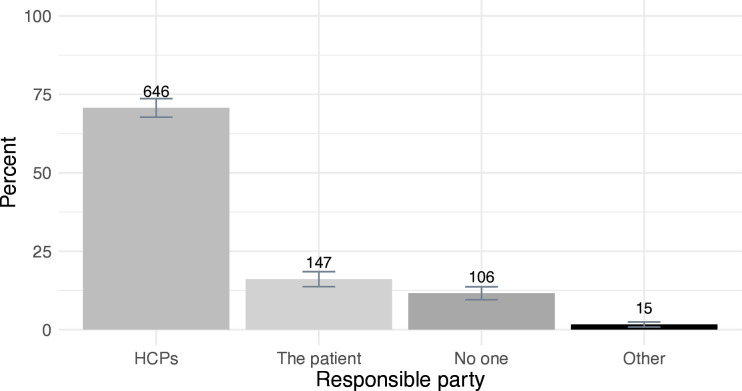
Proportion of respondents ascribing ultimate responsibility for informing at-risk relatives to healthcare providers (HCPs) (grey), the patient (light grey), no one (dark grey) or other (black).

When prompted on which party participants believed should have the ultimate responsibility for informing ARRs, 71% (n=646, p<0.001) ascribed this responsibility to HCPs, while 16% thought that the patient should have this responsibility and 12% believed that no one should (figure 2). The tendency to ascribe ultimate responsibility to HCPs was also present when respondents were stratified into different subgroups (online supplemental table 3).

### Legal obligation to inform ARRs

**Figure 3:**
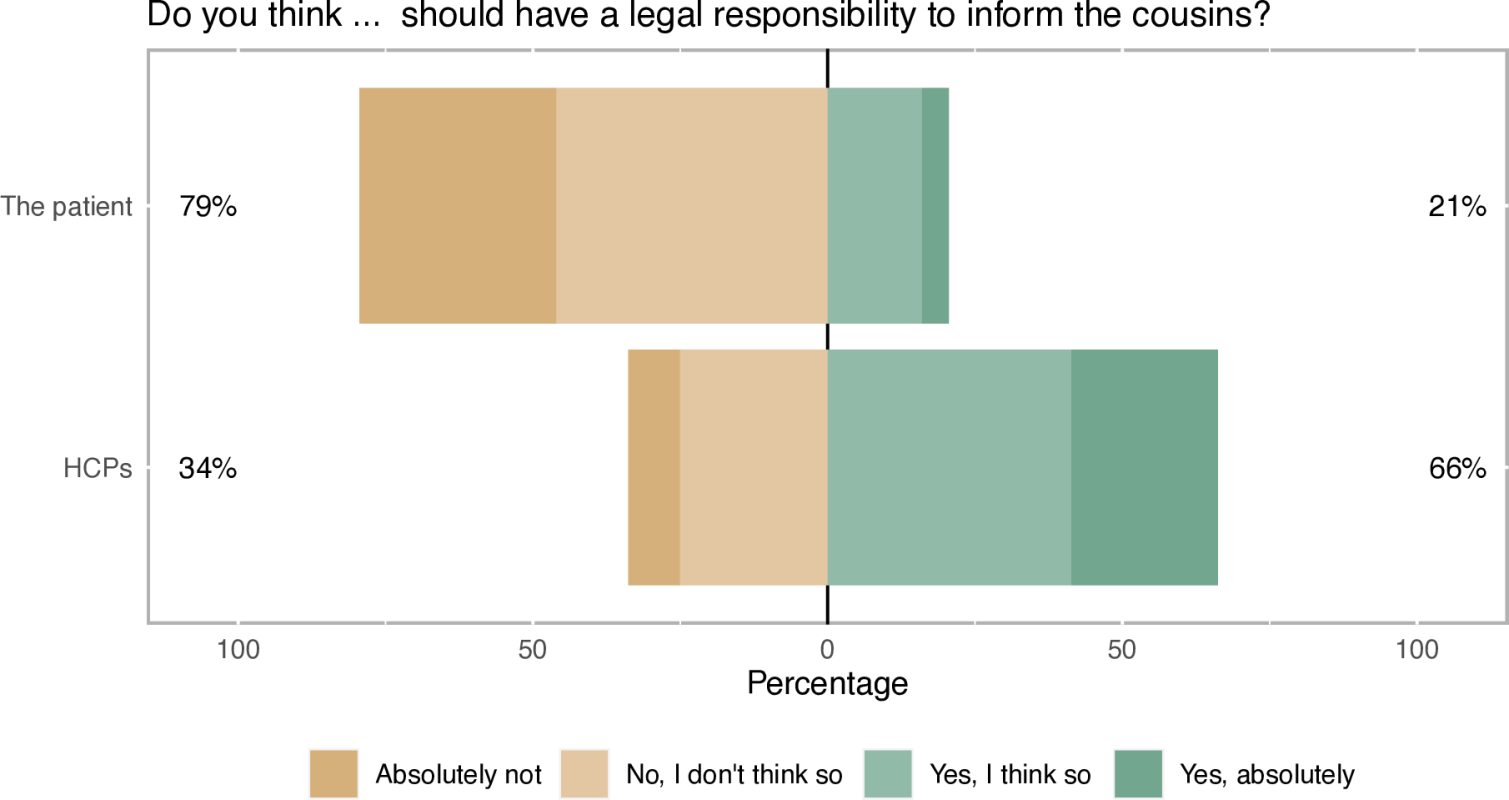
Attitudes on whether the patient and/or healthcare providers (HCPs) should have a legal responsibility to inform at-risk relatives.

In univariable analysis, 21% thought that the patient should have a legal obligation to inform ARRs while 66% thought that HCPs should have such a duty (figure 3). When cross-tabulating these questions, 48%, (n=440) thought only HCPs should have a legal obligation, whereas 31% (n=286) thought that no one should have this duty (online supplemental figure S2). The opinion that HCPs should have a legal obligation to inform ARRs was more pronounced among women than men (p=0.003) and among younger as compared with older respondents (p<0.001). Among those who would like to be informed about a potential risk for colorectal cancer, and those who wanted their relatives to be informed about such risk, a significantly larger proportion ascribed a legal responsibility to the patient, as well as HCPs, compared with those who did not want to be informed, or did not want their relatives to be informed (online supplemental table 4).

### Should the HCPs inform ARRs against the patient’s will?

**Figure 4:**
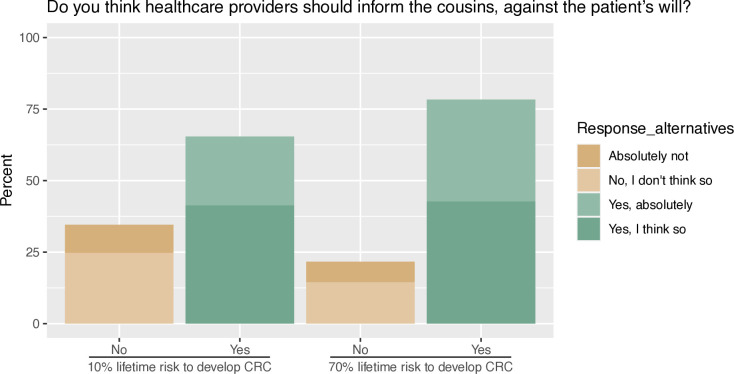
Attitudes on whether healthcare providers should inform at-risk relatives against the will of the patient at different lifetime risk for colorectal cancer (CRC).

A majority of respondents thought that HCPs should inform the ARRs against the patient’s will if the ARRs’ risk of developing colorectal cancer was moderate or high (65% if moderate and 78% if the risk was high) (figure 4). When stratified into subgroups, this preference was more pronounced for younger than older individuals and for those without children compared with those who do have children (online supplemental table S5). Among those who would like to be informed about a potential risk for colorectal cancer, and those who wanted their relatives to be informed about such risk, a significantly larger proportion thought that HCP should inform ARRs against the patients’ will, as compared with those who did not want to be informed or did not want their relatives to be informed (p<0.001).

## Discussion

In Sweden, the current standard practice is that HCPs support patients in informing ARRs, while leaving it to the patient to do the actual informing. The support offered includes genetic counselling and the provision of family letters. This practice is in line with most guidelines internationally, which emphasise the patient’s role in communication with their ARRs.^8^ Our results, however, indicate that public opinion would support a reversal of these roles, with HCPs taking the lead in ensuring that ARRs are informed.

That 51% of respondents held that both the index patient and HCPs have a moral responsibility to inform may indicate an expectation of shared responsibility and cooperation between the parties (as happens under current practice). These results are in line with findings from a qualitative focus group study with the Swedish public where participants voiced a desire that risk disclosure to ARRs should be a shared responsibility between the index patient and HCPs.^19^

The gap between public attitudes and standard practice is even larger when it comes to ultimate responsibility—who has the final and most important responsibility (with 71% responding that HCPs should have that responsibility, while only 16% of respondents placed it with the patient). This raises the question of whether alternative approaches to family communication granting HCPs a more active role in the communication process should be considered. Meta-analysis, based mainly on observational studies, indicates that the current praxis of family-mediated risk disclosure is not very effective, leading to an uptake of genetic counselling among ARRs of about 35% (95% CI, 24% to 48%).^24^

One way for HCPs to take more responsibility is to make sure that ARRs are informed by actively informing them. Previous interventions with HCP-led disclosure (also known as direct contact) increased the rates of cascade genetic counselling to 63% (95% CI, 49% to 75%).^24^ Empirical research of public attitudes indicate that there is support for HCP-led risk disclosure to ARRs.^1922 25^,^27^ Among patients and ARRs in families with hereditary cancer syndrome, HCP-led risk disclosure is viewed as an alternative pathway for information dissemination and when there is a distant or strained family relationship it may be the preferred or only possible mode of risk disclosure.^28 29^

On the other hand, it should be recognised that there is limited data from randomised studies on the effectiveness of HCP-led direct contact. When being implemented in a real-world clinical setting in the Netherlands, a proactive approach—including direct contact with ARRs—did not increase the uptake of testing as compared with the previous (family-mediated) risk disclosure practice.^30^ An evaluation of the Danish Lynch registry model of sending direct letters to ARRs shows that 1535 of 6507 (23.6%) ARRs were not contacted by the registry even if they were untested, indicating that HCP-led risk disclosure requires resources and a sustainable model to be successful.^31^ A direct approach, where the HCPs directly contact ARRs, also raises concerns about patients’ and ARRs’ possible (negative) reactions, as well as concerns around respect for the patient’s right to privacy and their ARRs’ right not to know. Furthermore, there are concerns about increased workload for HCPs and other practical obstacles, particularly given the lack of regulatory clarity, as evidenced by empirical research.^32^

Another indication that the public holds HCPs to be primarily responsible is the fact that over three times more respondents expressed that HCPs, as opposed to patients, should have a legal obligation to inform ARRs. However, it should be noted that these numbers may to some extent be explained by the perception that public institutions and individual behaviour differ in how they are best influenced—while social norms may be sufficient to promote pro-social individual behaviour, institutions are formal entities that need to be regulated. That fewer respondents ascribed legal as opposed to moral responsibility to both parties—patient and HCPs—may be explained by the fact that people may generally be more willing to assign moral rather than legal responsibility, since the latter implies possible legal enforcement.

Swedish legislation clearly states that the patient’s consent is mandatory for disclosing any information about the patient to ARRs. Thus, if the patient does not consent to share information about her genetic condition with the ARRs, the HCPs are currently not allowed to breach confidentiality. The communication of hereditary risk information within families is more explicitly addressed in the legal framework in other countries.^17^ For example, legislation in France places a legal obligation on patients to inform ARRs (either directly or through their HCPs) and legislation in Australia permits clinicians to inform ARRs even without the consent of the patient.^33 34^ In the UK, the court case ABC v St George’s Healthcare NHS Trust and others impose coexisting duties towards both the patient and the ARRs and suggest a legal obligation on HCPs to weigh the interest of patients with those of their ARRs.^35^

While cases of active non-disclosure represent a minority of cases,^36 37^ a majority of respondents in our survey endorse a responsibility for HCPs to inform ARRs even in cases where the patient explicitly objects to disclosure. Our data contrast with findings from a survey conducted in Israel where only about 20% thought HCPs should inform ARRs at risk of hereditary cancer even without patient consent.^38^ How might we interpret the majority view of our respondents? We see at least two options. One is the idea put forth in the literature that genetic information is familial in nature and as such does not belong to any individual person or patient.^39^,^42^ On that line of thinking, there is no moral basis for a legal right of patients to withhold information about ARRs potential genetic risk. Another interpretation is that the respondents believe that the ARRs’ interest in receiving the information overrides the patient’s right to confidentiality, which should therefore not be protected by law. Regardless of how exactly we should interpret the public’s inclination to endorse information to ARRs against the patient’s will, it is another indication that the public wants the HCPs to take an active role in informing ARRs or making sure they are informed.

Differences observed between subgroups as divided by sex, age, educational level, having children and cancer history were relatively modest. The fact that younger people were more prone to ascribe moral responsibility to HCPs may indicate a generational shift. The only subgroups that diverge quite substantially from the majority are those who did not themselves want to be informed, and those who did not want their relatives to be informed. These subgroups are much less prone to ascribe moral responsibility, especially to the patient. This is unsurprising—if one does not want to be informed or one’s relatives to be informed, it makes sense to reject the idea that anyone should be responsible for informing.

It is important to note that HCPs can take a more active role while still being respectful of other rights and interests. Patients may or may not have a moral right to refuse disclosure of the information (our results indicate most think they do not). ARRs may or may not have a moral right not to know about their genetic risks (previous data^22 25 43^ show that about 90% of the public want such unsolicited information). These possible rights are part of the moral terrain to be traversed by HCPs in living up to their responsibility to inform, if they have one (which our results indicate the public thinks they do).

It is also important to note that taking responsibility for informing ARRs includes interacting with other parties who are needed to fulfil this responsibility. For instance, HCPs may be dependent on the index patient’s willingness to share information that enables the identification of ARRs and their contact details. Our survey did not explore participants views on moral requirements to support or enable the provision of information by another party. Hence, it is quite possible that respondents who said that either the patient or HCP lacks a responsibility to inform still hold that they have an obligation to support the other party’s ability to inform.

The attitude that the healthcare system—and the healthcare professionals as actors within it—should take responsibility for informing ARRs about their potential hereditary cancer risk may indicate that there is a general expectation that if one is at increased risk of cancer, then one should be informed about this (if preventive measures are available). If that is true, it seems that good reason would be required for not delivering on this expectation—especially considering the improved health outcomes that could only be realised by disseminating this information. Practical problems to do with workload and lack of regulation would need to be addressed on the path towards creating a sustainable risk disclosure model.

### Methodological considerations

We surveyed a random sample of the Swedish adult population for their attitudes on a hypothetical clinical situation involving the disclosure of a hereditary cancer risk to ARRs. We believe that the earlier parts of the survey made the respondents familiar with the topic and so more prepared to give responses about the moral and legal issues that we present here.

The hypothetical situation involves informing a patient’s third-degree ARRs (cousins) when the patient is unwilling to get in touch with them (because they have not spoken for 20 years). A description of a non-problematic situation, for example, one of informing a sibling with which the patient is in regular contact, would very likely have yielded different answers. However, our hypothetical situation is designed to be rather typical of difficult situations, where ‘lost contact’ may be a barrier for the patient to disseminate information. Some situations are more problematic than this one. In our hypothetical case, there are no conflicts or other extreme obstacles, there is just an absence of an established and active relationship, often referred to as ‘lost contact’ in the counselling situation. Whereas active non-disclosure is rare,^36 37^ ‘lost contact’ is a barrier often raised by patients as a reason for passive non-disclosure.^5^

Limitations include the use of a hypothetical scenario. While public attitudes may reflect underlying values, they may not directly translate to attitudes towards a similar real-life experience.^44^ The data was collected a few years ago, and there is a possibility of a shift in attitudes since then, especially since younger respondents are more prone to ascribe responsibility to HCPs. We therefore plan to repeat the questionnaire. Another limitation is that even though we stratified the invited sample to reflect the general public, we have an over-representation of respondents at a higher age, with higher education and those born in Sweden. As a result, generalisability of our findings to other groups and cultural contexts are limited.

## Conclusion

Our data shows that the Swedish public think HCPs have a moral responsibility to inform ARRs about an increased risk of hereditary colorectal cancer. The public also ascribe the same moral responsibility to patients, but to a lower degree. When asked about which party should have the ultimate responsibility for risk disclosure, a majority (n=646, 71%, p<0.001) thought this belonged to HCPs. A majority of respondents also thought that HCPs should have a legal obligation for informing ARRs, and a majority believe that they should do so even against the patient’s expressed wishes. It seems clear that the Swedish public reject the current clinical practice of placing the moral responsibility to inform ARRs with the patient. These public expectations should be considered when planning for future care pathways for patients with hereditary cancer and their ARRs.

## supplementary material

10.1136/bmjopen-2024-089237 online supplemental file 1

## Data Availability

Data are available upon reasonable request.
