# Supplementary material for: Who has the responsibility to inform relatives at risk of hereditary cancer? A population-based survey in Sweden
Source: BMJ Open. 2024 Nov 27;14(11):e089237. doi: 10.1136/bmjopen-2024-089237 (PMC11603682; doi:10.1136/bmjopen-2024-089237)
Supplement: online supplemental file 1 [file bmjopen-14-11-s001.docx]

Supplementary information

[**Supplementary table S1.** Translated questions and response options with descriptive statistics. 2](#_Toc180680707)

[**Supplementary table S2.** Subgroup analysis of respondents´attitudes on whether the patient and/or healthcare providers (HCPs) has/have a moral responsibility to inform at-risk relatives. 4](#_Toc180680708)

[**Supplementary table S3.** Subgroup analysis of respondents’ attitudes on which party should be ascribed ultimate responsibility to inform at-risk relatives. 6](#_Toc180680709)

[**Supplementary table S4.** Subgroup analysis of respondents’ attitudes on whether the patient and/or healthcare providers (HCPs) should have a legal obligation to inform at-risk relatives. 7](#_Toc180680710)

[**Supplementary table S5.** Subgroup analysis of respondents’ attitudes on whether health care providers (HCPs) should inform at-risk relatives against the patient´s will, at different levels of lifetime CRC-risk. 9](#_Toc180680711)

[**Supplementary table S6.** Original questionnaire (in Swedish). 11](#_Toc180680712)

[**Supplementary figure S1.** Respondents ascribing moral responsibility to inform the relatives to both the patient and healthcare providers (HCPs) (purple), only to healthcare providers (blue), only to the patient (pink) or none (grey). 14](#_Toc180680713)

[**Supplementary figure S2**. Respondents who thought a legal obligation to inform the relatives should be imposed on both the patient and healthcare providers (HCPs) (purple), only on HCPs (blue), only on the patient (pink) or none (grey). 14](#_Toc180680714)

## **Supplementary table S1.** Translated questions and response options with descriptive statistics.

| Introduction: The following section concerns your thoughts on how hereditary cancer risk information should be handled. In some families there is an increased risk of cancer. Affected relatives can be offered health checks in order to early detect and remove early stages of cancer. The chances of being cured increase greatly if the cancer is discovered early on. We would like you to imagine being part of six scenarios and answer the accompanying questions. The scenarios are all examples of situations that arise at cancer genetic units in Swedish clinical practice.  Scenario 5. Kim, 40 years old, has initiated a cancer genetic investigation because several of Kim´s relatives had colorectal cancer rather young. The investigation shows that Kim, Kim’s siblings and Kim’s cousins may have an increased risk of developing colorectal cancer. They can be offered regular colonoscopies. Kim informs the siblings, but has not spoken with the cousins for 20 years and does not want to contact them. | | | | |
| --- | --- | --- | --- | --- |
| **Question** | **Response options** | | | |
| q131 Do you think Kim has a moral responsibility to inform the cousins? | No, absolutely not | No, I don’t think so | Yes, I think so | Yes, absolutely |
| **Total: 914 responses** | n=107 (12%) | n=267 (29%) | n=320 (35%) | n=220 (24%) |
| q132 Do you think healthcare providers have a moral responsibility to inform the cousins? | No, absolutely not | No, I don’t think so | Yes, I think so | Yes, absolutely |
| **Total: 914 responses** | n=49 (5 %) | n=183 (20%) | n=357 (39%) | n=325 (36%) |
| q133 Who, in your opinion, should be ultimately responsible for informing the cousins? | Kim | Healthcare providers | Nobody | Other |
| **Total: 914 responses** | n=147, (16%) | n=646, (71%) | n=106  (12%) | n=15  (2%) |
| q134 Do you think Kim should have a legal obligation to inform the cousins? | No, absolutely not | No, I don’t think so | Yes, I think so | Yes, absolutely |
| **Total: 914 responses** | n=305  (33 %) | n=421 (46%) | n=146 (16%) | n=42  (5 %) |
| q135 Do you think healthcare providers should have a legal obligation to inform the cousins? | No, absolutely not | No, I don’t think so | Yes, I think so | Yes, absolutely |
| **Total: 914 responses** | n=80  (9%) | n=229 (25%) | n=378 (41%) | n=227 (25%) |
| **Question** | **Response options** | | | |
| q136 Kim does not want to inform the cousins and does not want to let healthcare providers do it either. Do you think healthcare providers should inform the cousins against Kim’s will that they may have a doubled lifetime risk of developing colorectal cancer (around 10 percent compared to the standard 5 percent)? | No, absolutely not | No, I don’t think so | Yes, I think so | Yes, absolutely |
| **Total: 914 responses** | n=90 (10%) | n=226 (25%) | n=378 (41%) | n=220 (24%) |
| q137 Kim does not want to inform the cousins and does not want to let healthcare providers do it either. Do you think healthcare providers should inform the cousins against Kim’s will that they may have a doubled lifetime risk of developing colorectal cancer (around 70 percent compared to the standard 5 percent)? | No, absolutely not | No, I don’t think so | Yes, I think so | Yes, absolutely |
| **Total: 914 responses** | n=66 (7%) | n=132 (14%) | n=391 (43%) | n=325 (36%) |
| **Scenario 1.** Your relative Kit has initiated a family investigation at a cancer genetic unit. The investigation shows that several individuals in your family may have a doubled risk of developing colorectal cancer sometime during their life (around 10 percent lifetime risk compared to average 5 percent). Relatives at risk can be offered colonoscopies every fifth year to early detect, or remove, early stages of cancer. | | | | |
| q99 Would you like to be informed about the family investigation done by Kit? | No, absolutely not | No, I don’t think so | Yes, I think so | Yes, absolutely |
| **Total: 914 responses** | 10  (1%) | 76 (8%) | 357 (39%) | 471 (52%) |
| **Scenario 2.** You have initiated a family investigation at a cancer genetic unit. The investigation shows that several individuals in your family may have a doubled risk of developing colorectal cancer (around 10 percent lifetime risk compared to average 5 percent). Affected individuals can be offered colonoscopies every fifth year to early detect, or remove, early stages of cancer. | | | | |
| Would you want your relatives to be informed about the family investigation you have done? | No, absolutely not | No, I don’t think so | Yes, I think so | Yes, absolutely |
| **Total: 912 responses** | 14 (2%) | 55 (6%) | 347 (38%) | 496 (54%) |

## **Supplementary table S2.** Subgroup analysis of respondents´attitudes on whether the patient and/or healthcare providers (HCPs) has/have a moral responsibility to inform at-risk relatives.

|  |  | **The patient** | |  | **HCPs** | |  |
| --- | --- | --- | --- | --- | --- | --- | --- |
|  | Subgroup | Yes | No | P-value Chi2 | Yes | No | P-value Chi2 |
| **Total** | - | 540 (59.1%) | 374  (40.9%) |  | 682 (74.6%) | 232 (25.4%) |  |
|  |  |  |  |  |  |  |  |
| **Gender** | Women | 263    (60.7%) | 170 (60.7%) |  | 313 (72.3%) | 120 (27.7%) |  |
|  | Men | 277 (57.6%) | 204 (42.4) | 0.37 | 369  (76.7%) | 112 (23.3%) | 0.14 |
|  |  |  |  |  |  |  |  |
| **Age** | 18-29 | 75 (61.0%) | 48 (39.0%) |  | 107 (87.0%) | 16 (13.0%) |  |
|  | 30-39 | 74 (54.0%) | 63 (46.0%) |  | 114  (83.2%) | 23 (16.8%) |  |
|  | 40-49 | 90 (57.3%) | 67 (42.7%) |  | 112 (71.3%) | 45 (28.7%) |  |
|  | 50-59 | 85 (56.7%) | 65 (43.3%) |  | 114  (76.0%) | 36 (24.0%) |  |
|  | 60-69 | 122 (63.2%) | 71 (36.8%) |  | 134 (69.4%) | 59 (30.6%) |  |
|  | 70-74 | 94 (61.0%) | 60 (39.0%) | 0.58 | 101  (65.6%) | 53 (34.4%) | <0.001 |
|  |  |  |  |  |  |  |  |
| **Education** | Lower | 227 (62.0%) | 139 (38.0%) |  | 268 (73.2%) | 98 (26.8%) |  |
|  | Middle | 154 (52.9%) | 137 (47.1%) |  | 215 (73.9%) | 76  (26.1%) |  |
|  | Higher | 155 (61.5%) | 97 (38.5%) | 0.04 | 195 (77.4%) | 57  (22.6%) | 0.48 |
|  |  |  |  |  |  |  |  |
| **Country of birth** | Sweden | 487 (57.8%) | 356 (42.2%) |  | 624  (74.0%) | 219 (26.0%) |  |
|  | Other | 53 (74.6%) | 18 (25.4%) | 0.008 | 58 (81.7%) | 13 (18.3%) | 0.20 |
|  |  |  |  |  |  |  |  |
| **Children** | Yes | 358 (59.9%) | 240 (40.1%) |  | 430 (71.9%) | 168 (28.1%) |  |
|  | No | 178 (57.2%) | 133 (42.8%) | 0.49 | 248 (79.7%) | 63 (20.3%) | 0.01 |
|  |  |  |  |  |  |  |  |
| **Cancer history** | Yes | 53 (66.3%) | 27 (33.8%) |  | 60 (75.0%) | 20 (25.0%) |  |
|  | No | 484 (58.5%) | 344 (41.5%) | 0.22 | 617 (74.5%) | 211 (25.5%) | 1.00 |
|  |  |  |  |  |  |  |  |
| **Wants to be informed about a potential hereditary risk of CRC** | Yes | 513 (62.0%) | 315 (38.0%) |  | 645 (77.9%) | 183 (22.1%) |  |
|  | No | 27 (31.4%) | 59 (68.6%) | <0.001 | 37 (43.0%) | 49 (57.0%) | <0.001 |
|  |  |  |  |  |  |  |  |
| **Wants their relatives to be informed about a potential hereditary risk for CRC** | Yes | 525 (62.1%) | 320 (37.9%) |  | 653 (77.3%) | 192 (22.7%) |  |
|  | No | 15 (21.7%) | 54 (78.3%) | <0.001 | 29 (42.0%) | 40 (58.0%) | <0.001 |

## **Supplementary table S3.** Subgroup analysis of respondents’ attitudes on which party should be ascribed ultimate responsibility to inform at-risk relatives.

|  |  | **Responsible party** | | | |
| --- | --- | --- | --- | --- | --- |
|  | **Subgroup** | **HCPs** | **The patient** | **None** | **Other** |
| **Total** | - | 646 (70.7%) | 147 (16.1%) | 106 (11.6%) | 15 (1.6%) |
|  |  |  |  |  |  |
| **Gender** | Women | 300 (69.3%) | 69 (15.9%) | 55 (12.7%) | 9 (2.1%) |
|  | Men | 346 (71.9%) | 78 (16.2%) | 51 (10.6%) | 6 (1.2%) |
|  |  |  |  |  |  |
| **Age** | 18-29 | 100 (81.3%) | 17 (13.8%) | 5 (4.1%) | 1 (0.8%) |
|  | 30-39 | 106 (77.4%) | 17 (12.4%) | 11 (8.0%) | 3 (2.2%) |
|  | 40-49 | 106 (67.5%) | 26 (16.6%) | 21 (13.4%) | 4 (2.5%) |
|  | 50-59 | 104 (69.3%) | 23 (15.3%) | 20 (13.3%) | 3 (2.0%) |
|  | 60-69 | 130 (67.4%) | 34 (17.6%) | 27 (14.0%) | 2 (1.0%) |
|  | 70-74 | 100 (64.9%) | 30 (19.5%) | 22 (14.3%) | 2 (1.3%) |
|  |  |  |  |  |  |
| **Education** | Lower | 256 (69.9%) | 60 (16.4%) | 45 (12.3%) | 5 (1.4%) |
|  | Middle | 208 (71.5%) | 41 (14.1%) | 36 (12.4%) | 6 (2.1%) |
|  | Higher | 178 (70.6%) | 45 (17.9%) | 25 (9.9%) | 4 (1.4%) |
|  |  |  |  |  |  |
| **Country of birth** | Sweden | 597 (70.8%) | 133 (15.8%) | 99 (11.7%) | 14 (1.7%) |
|  | Other | 49 (75.4%) | 14 (12.3%) | 7 (10.8%) | 1 (1.5%) |
|  |  |  |  |  |  |
| **Children** | Yes | 415 (69.4%) | 95 (15.9%) | 78 (13.0%) | 10 (1.7%) |
|  | No | 227 (73.0%) | 51 (16.4%) | 28 (9.0%) | 5 (1.6%) |
|  |  |  |  |  |  |
| **Cancer history** | Yes | 54 (67.5%) | 14 (17.5%) | 12 (15.0%) | 0 |
|  | No | 589 (71.1%) | 131 (15.8%) | 93 (11.2%) | 15 (1.8%) |
|  |  |  |  |  |  |
| **Wants to be informed about a potential hereditary risk for CRC** | Yes | 603(72.8%) | 127(15.3%) | 84 (10.1%) | 14 (1.7%) |
|  | No | 43 (50.0%) | 20 (23.3%) | 22 (25.6%) | 1 (1.2%) |
|  |  |  |  |  |  |
| **Wants their relatives to be informed about a potential hereditary risk for CRC** | Yes | 608 (72.0%) | 135 (16.0%) | 87 (10.3%) | 15 (1.8%) |
|  | No | 38 (55.1%) | 12 (17.4%) | 19 (27.5%) | 0 |

## **Supplementary table S4.** Subgroup analysis of respondents’ attitudes on whether the patient and/or healthcare providers (HCPs) should have a legal obligation to inform at-risk relatives.

|  |  | **The patient** | |  | **HCPs** | |  |
| --- | --- | --- | --- | --- | --- | --- | --- |
|  | Subgroup | Yes | No | P-value Chi2 | Yes | No | P-value Chi2 |
| **Total** | - | 188  (20.6%) | 726  (79.4%) |  | 605  (66.2%) | 309  (33.8%) |  |
|  |  |  |  |  |  |  |  |
| **Gender** | Women | 95 (21.9%) | 338 (78.1%) |  | 340  (70.7%) | 141  (29.3%) |  |
|  | Men | 93  (19.3%) | 388 (80.7%) | 0.37 | 265  (61.2%) | 168  (38.8%) | 0.003 |
|  |  |  |  |  |  |  |  |
| **Age** | 18-29 | 32  (26.0%) | 91 (74.0%) |  | 98  (70.7% | 25  (29.3%) |  |
|  | 30-39 | 24  (17.5%) | 113 (82.5%) |  | 107  (78.1%) | 30  (21.9%) |  |
|  | 40-49 | 33  (21.0%) | 124 (79.0%) |  | 103  (65.6%) | 54  (34.4%) |  |
|  | 50-59 | 35  (23.3%) | 115 (76.7%) |  | 93  (62.0%) | 57  (38.0%) |  |
|  | 60-69 | 33  (17.1%) | 160 (82.9%) |  | 111  (57.5%) | 82  (42.5%) |  |
|  | 70-74 | 31 (20.1%) | 123 (79.9%) | 0.39 | 93  (60.4%) | 61  (39.6%) | <0.001 |
|  |  |  |  |  |  |  |  |
| **Education** | Lower | 85 (23.2%) | 281 (76.8%) |  | 240  (65.6%) | 126  (34.4%) |  |
|  | Middle | 56 (19.2%) | 235 (80.8%) |  | 192  (66.0%) | 99  (34.0%) |  |
|  | Higher | 43 (17.1%) | 209 (82.9%) | 0.15 | 168  (66.7%) | 84  (33.3%) | 0.96 |
|  |  |  |  |  |  |  |  |
| **Country of birth** | Sweden | 163 (19.3%) | 680 (80.7%) |  | 554  (65.7%) | 289  (34.3%) |  |
|  | Other | 25 (35.2%) | 46 (64.8%) | 0.003 | 51  (71.8%) | 20  (28.2%) | 0.36 |
|  |  |  |  |  |  |  |  |
| **Children** | Yes | 117 (19.6%) | 481 (80.4%) |  | 375  (62.7%) | 223  (37.3%) |  |
|  | No | 70 (22.5%) | 241 (77.5%) | 0.34 | 226  (72.7%) | 85  (27.3%) | 0.003 |
|  |  |  |  |  |  |  |  |
| **Cancer history** | Yes | 19 (23.8%) | 61 (76.3%) |  | 48  (60.0%) | 32  (40.0%) |  |
|  | No | 167 (20.2%) | 661 (79.8%) | 0.54 | 551  (66.5%) | 277  (33.5%) | 0.29 |
|  |  |  |  |  |  |  |  |
| **Wants to be informed about a potential hereditary risk for CRC** | Yes | 181 (21.9%) | 647 (78.1%) |  | 576  (69.6%) | 252  (30.4%) |  |
|  | No | 7 (8.1%) | 79 (91.9%) | 0,004 | 29  (33.7%) | 57  (66.3%) | <0.001 |
|  |  |  |  |  |  |  |  |
| ***Wants their relatives to be informed about a potential hereditary risk for CRC** | Yes | 185 (21.9%) | 660 (78.1%) |  | 583  (69.0%) | 262  (31.0%) |  |
|  | No | 3 (4.3%) | 66 (95.7%) | <0.001 | 22  (31.9%) | 47  (68.1%) | <0.001 |

## **Supplementary table S5.** Subgroup analysis of respondents’ attitudes on whether health care providers (HCPs) should inform at-risk relatives against the patient´s will, at different levels of lifetime CRC-risk.

|  |  | **10% lifetime CRC-risk** | |  | **70% lifetime CRC-risk** | |  |
| --- | --- | --- | --- | --- | --- | --- | --- |
|  | Subgroup | Yes | No | P-value Chi2 | Yes | No | P-value Chi2 |
| **Total** | - | 598 (65.4%) | 316 (34.6%) |  | 716 (78.3%) | 198 (21.7%) |  |
|  |  |  |  |  |  |  |  |
| **Gender** | Women | 265 (61.2%) | 168 (38.8%) |  | 329 (76.0%) | 104 (24.0%) |  |
|  | Men | 333 (69.2%) | 148 (30.8%) | 0.013 | 387 (80.5%) | 94 (19.5%) | 0.12 |
|  |  |  |  |  |  |  |  |
| **Age** | 18-29 | 98 (79.7%) | 25 (20.3%) |  | 109 (88.6%) | 14 (11.4%) |  |
|  | 30-39 | 110 (80.3%) | 27 (19.7%) |  | 121 (88.3%) | 16 (11.7%) |  |
|  | 40-49 | 108 (68.8%) | 49 (31.2%) |  | 131 (83.4%) | 26 (16.6%) |  |
|  | 50-59 | 85 (56.7%) | 65 (43.3%) |  | 113 (75.3%) | 37 (24.7%) |  |
|  | 60-69 | 114 (59.1%) | 79 (40.9%) |  | 141 (73.1%) | 52 (26.9%) |  |
|  | 70-74 | 83 (53.9%) | 71 (46.1%) | <0.001 | 101 (65.6%) | 53 (34.4%) | <0.001 |
|  |  |  |  |  |  |  |  |
| **Education** | Lower | 242 (66.1%) | 124 (33.9%) |  | 280 (76.5%) | 86 (23.5%) |  |
|  | Middle | 189 (64.9%) | 102 (35.1%) |  | 221 (75.9%) | 70 (24.1%) |  |
|  | Higher | 163 (64.7%) | 89 (35.3%) | 0.92 | 210 (83.3%) | 42 (16.7%) | 0.07 |
|  |  |  |  |  |  |  |  |
| **Country of birth** | Sweden | 545 (64.7%) | 298 (35.3%) |  | 658 (78.1%) | 185 (21.9%) |  |
|  | Other | 53 (74.6%) | 18 (25.4%) | 0.12 | 58 (81.7%) | 13 (18.3%) | 0.57 |
|  |  |  |  |  |  |  |  |
| **Children** | Yes | 365 (61.0%) | 233 (39.0%) |  | 451 (75.4%) | 147 (24.6%) |  |
|  | No | 230 (74.0%) | 81 (26.0%) | <0.001 | 261 (83.9%) | 50 (16.1%) | 0.004 |
|  |  |  |  |  |  |  |  |
| **Cancer history** | Yes | 50 (62.5%) | 30 (37.5%) |  | 66 (82.5%) | 14 (17.5%) |  |
|  | No | 543 (65.6%) | 285 (34.4%) | 0.67 | 645 (77.9%) | 183 (22.1%) | 0.42 |
|  |  |  |  |  |  |  |  |
| **Wants to be informed about a potential hereditary risk for CRC** | Yes | 573 (69.2%) | 255 (30.8%) |  | 677 (81.8%) | 151 (18.2%) |  |
|  | No | 25 (29.1%) | 61 (70.9%) | <0.001 | 39 (45.3%) | 47 (54.7%) | <0.001 |
|  |  |  |  |  |  |  |  |
| **Wants their relatives to be informed about a potential hereditary risk for CRC** | Yes | 576 (68.2%) | 269 (31.8%) |  | 687 (81.3%) | 158 (18.7%) |  |
|  | No | 22 (31.9%) | 47 (68.1%) | <0.001 | 29 (42.0%) | 40 (58.0%) | <0.001 |

## **Supplementary table S6.** Original questionnaire (in Swedish).

Start of Block: s5: scenario 5

q130 Scenario 5. Kim, 40 år, har startat en cancergenetisk utredning eftersom flera av Kims släktingar haft tjocktarmscancer i unga år. Utredningen visar att Kim, Kims syskon och Kims kusiner kan ha en ökad risk att utveckla tjocktarmscancer. De kan erbjudas regelbundna tarmundersökningar. Kim informerar sina syskon, men har inte pratat med sina kusiner på 20 år och vill inte höra av sig till dem.

q131 Tycker du att Kim har ett moraliskt ansvar att informera kusinerna?

- Nej, absolut inte (1)
- Nej, jag tror inte det (2)
- Ja, jag tror det (3)
- Ja, absolut (4)

q132 Tycker du att *sjukvården* har ett moraliskt ansvar att informera kusinerna?

- Nej, absolut inte (1)
- Nej, jag tror inte det (2)
- Ja, jag tror det (3)
- Ja, absolut (4)

q133 Vem tycker du ska vara ytterst ansvarig att informera kusinerna?

- Kim (1)
- Sjukvården (2)
- Ingen (3)
- Annan: (4) ________________________________________________

q134 Tycker du att Kim borde ha en laglig skyldighet att informera kusinerna?

- Nej, absolut inte (1)
- Nej, jag tror inte det (2)
- Ja, jag tror det (3)
- Ja, absolut (4)

q135 Tycker du att *sjukvården*borde ha en laglig skyldighet att informera kusinerna?

- Nej, absolut inte (1)
- Nej, jag tror inte det (2)
- Ja, jag tror det (3)
- Ja, absolut (4)

q136 Kim vill inte informera kusinerna själv, och vill heller inte låta sjukvården göra det. Tycker du att *sjukvården* ska informera kusinerna mot Kims vilja om att de kan ha en fördubblad risk att någon gång i livet insjukna i tjocktarmscancer (cirka 10 procent mot normala 5 procent)?

- Nej, absolut inte (1)
- Nej, jag tror inte det (2)
- Ja, jag tror det (3)
- Ja, absolut (4)

q137 Kim vill inte informera kusinerna själv, men vill inte heller låta sjukvården göra det. Tycker du att *sjukvården* ska informera kusinerna mot Kims vilja om att de kan ha en starkt ökad risk att någon gång i livet insjukna i tjocktarmscancer (cirka 70 procent mot normala 5 procent)?

- Nej, absolut inte (1)
- Nej, jag tror inte det (2)
- Ja, jag tror det (3)
- Ja, absolut (4)

q138 Om *sjukvården* ska informera kusinerna, vad tycker du den första informationen ska innehålla?

- Att en utredning har gjorts och att de kan höra av sig om de vill veta mer (1)
- Att en utredning har gjorts och att de har en ökad risk att insjukna i tjocktarmscancer (2)
- Annat: (3) ________________________________________________

q139 Hur tycker du att *sjukvården* i så fall ska ge kusinerna denna information?

- Via videosamtal (1)
- Via brev (2)
- Via telefonsamtal (3)
- Via e-post (4)
- Via SMS (5)
- Via inloggning på ”Mina vårdkontakter”, 1177 Vårdguiden (6)
- Annat: (7) ________________________________________________

q140 Om du har några kommentarer till Scenario 5 får du gärna lämna dem här:

________________________________________________________________

________________________________________________________________

________________________________________________________________

________________________________________________________________

________________________________________________________________

End of Block: s5: scenario 5

Comment: The Swedish word "sjukvården" refers to the part of society that delivers health care services. It may refer to either the health care system as an institution, or the individuals who deliver health care as health care professionals, or both. We have used "healthcare providers" as a translation of the Swedish word ”sjukvården”.

## **Supplementary figure S1.** Respondents ascribing moral responsibility to inform the relatives to both the patient and healthcare providers (HCPs) (purple), only to healthcare providers (blue), only to the patient (pink) or none (grey).

## **Supplementary figure S2**. Respondents who thought a legal obligation to inform the relatives should be imposed on both the patient and healthcare providers (HCPs) (purple), only on HCPs (blue), only on the patient (pink) or none (grey).
