# Supplementary material for: Who has the responsibility to inform relatives at risk of hereditary cancer? A population-based survey in Sweden
Source: BMJ Open. 2024 Nov 27;14(11):e089237. doi: 10.1136/bmjopen-2024-089237 (PMC11603682; doi:10.1136/bmjopen-2024-089237)
Supplement: Supplementary file 2 [file bmjopen-2024-089237.reviewer_comments.pdf]

PEER REVIEW HISTORY

BMJ Open publishes all reviews undertaken for accepted manuscripts. Reviewers are asked to complete a checklist review form and are provided with free text boxes to elaborate on their assessment. These free text comments are reproduced below.

ARTICLE DETAILS

Title (Provisional)

Who has the responsibility to inform relatives at risk of hereditary cancer? A population-based survey in Sweden.

Authors

Grill, Kalle; Phillips, Amicia; Numan Hellquist, Barbro; Rosén, Anna

---

VERSION 1 - REVIEW

**Reviewer**	**1**
**Name**	**Tiller, Jane**
**Affiliation**	**Monash University, Public Health Genomics**
**Date**	**24-Jul-2024**
**COI**	**N/A**

A well-designed and presented study that was a pleasure to read. Use of supplementary material to share full data was appreciated. Number of respondents and response rate were excellent.

My comments are below, by page because the line numbers are not continuous. I have used the page numbers in the top right rather than the bottom of the pages.

Page 6/34

Line 33-34: For non-Swedish readers, perhaps include a short description of how the invitation is sent. I was able to access a report from ref 13 (https://www.gu.se/sites/default/files/2020-04/TR_LORE_MP31.pdf) but it would do well to note the site address in the reference itself to guide readers. Ie do you have a government email inbox that everyone mandatorily checks, is it sent to personal email addresses etc?

Line 41: Is “the infrastructure” a government database/electronic health record of some sort, or a separate research database of some sort?

Line 42 onwards: I assume that the materials/questions etc were all in Swedish and have been translated to English for this manuscript? It might be worth saying so briefly.

Page 8/34

Line 6/7 (unclear): your % has no number in front of it (n=646)

Line 28-33: Was there any difference across age/gender/etc on this, or it was consistent across subgroup analysis?

Page 9/34

Line 31-33: what about Frey's 2022 systematic review (https://pubmed.ncbi.nlm.nih.gov/35960887/), which reviewed 87 studies and found, "Compared with the patient-mediated approach, direct relative contact resulted in significantly higher uptake of genetic counseling for all relatives (63% [95% CI, 49 to 75] v 35% [95% CI, 24 to 48]) and genetic testing for first-degree relatives (62% [95% CI, 49 to 73] v 40% [95% CI, 32 to 48])."?

Page 10/34

Line 33: For a more recent analysis of the Australian position, see https://pubmed.ncbi.nlm.nih.gov/32805446/

Line 39/40: I think you mean active NONdisclosure.

Page 12/24

Line 15-16: An important factor you have not noted is the trend in younger people towards viewing HCPs as having this obligation. Given you had an older population, and this trend still emerged, that is strong evidence that younger generations have a strong view. This may play out in your re-issue of the survey, but is worth briefly noting in this manuscript as well.

Line 27 (Conclusion): I wonder if you consider that any legal or structural changes should be considered by the Health Dept or Government? If so, rather than ending with a note that expectations should be considered, you could make a clear recommendation for what changes should be made.

**Reviewer**	**2**
**Name**	**Daack-Hirsch, Sandra**
**Affiliation**	**University of Iowa College of Nursing**
**Date**	**06-Aug-2024**
**COI**	**none**

I thought the article was well written and straightforward. I think there is room for improvement by adding a bit more description and clarity to the following:

1. Page 5. Please include any laws or legislation that govern HCP responsibility for disclosing genetic results to ARR here rather than in the discussion. Are there ramifications for the HCP if they do disclose results to ARR? Please describe how participants were randomly selected.

What is the online research infrastructure? It seems to me that there could be correlations among findings from the various scenarios and the 5th scenario reported here. I wonder if the discussion might be more robust if the authors included a discussion that considered the findings from the other

the scenarios as well. I think the discussion was a somewhat redundant of the findings.

Minor typos:

Page 7. A value is missing in the first paragraph, line 7.

Page 8. there are two commas, line 35.

VERSION 1 - AUTHOR RESPONSE

Reviewer: 1<br>Ms. Jane Tiller, Monash University	
Comments to the Author: A well-designed and presented study that was a pleasure to read. Use of supplementary material to share full data was appreciated. Number of respondents and response rate were excellent.	Thank you!
My comments are below, by page because the line numbers are not continuous. I have used the page numbers in the top right rather than the bottom of the pages.	
**Page 6/34**	
Line 33-34: For non-Swedish readers, perhaps include a short description of how the invitation is sent. I was able to access a report from ref 13 (https://www.gu.se/sites/default/files/2020-04/TR_LORE_MP31.pdf) but it would do well to note the site address in the reference itself to guide readers. Ie do you have a government email inbox that everyone mandatorily checks, is it sent to personal email addresses etc?	We have now clarified the section about the citizen panel by describing how the sample initially was recruited and added a reference to the Citizen web page (in English). Page 6, line 51-58; *"Participants were recruited through the digital research infrastructure Citizen Panel, hosted by the Laboratory of Opinion Research (LORE) at the University of Gothenburg, Sweden.[20] We invited a stratified sample of panelists that had previously been recruited to the Citizen Panel from a randomly selected sample of the Swedish Population Register Survey data by distributing an electronic questionnaire.[21]".*
Line 41: Is "the infrastructure" a government database/electronic health record of some sort, or a separate research database of some sort?	It is a separate research database held by the SOM Institute at Gothenburg University. We have now clarified the section about the

	research infrastructure, and added a reference to their webpage, please see above.
Line 42 onwards: I assume that the materials/questions etc were all in Swedish and have been translated to English for this manuscript? It might be worth saying so briefly.	Thanks for this comment. We have now added this information.
**Page 8/34**	
Line 6/7 (unclear): your % has no number in front of it (n=646)	Corrected
Line 28-33: Was there any difference across age/gender/etc on this, or it was consistent across subgroup analysis?	Thanks for this comment. We have now added a subgroup analysis of this question (Supplement Table 5) Please see added text on page 9, line 17 and onwards; *"When stratified into subgroups, this preference was more pronounced for younger than older individuals and for those without children compared to those who do have children (Supplementary table S5). Among those who would like to be informed about a potential risk for colorectal cancer, and those who wanted their relatives to be informed about such risk, a significantly larger proportion thought that HCP should inform ARRs against the patients will, as compared to those who did not want to be informed, or did not want their relatives to be informed (P<0.001)."*
**Page 9/34**	
Line 31-33: what about Frey's 2022 systematic review (https://pubmed.ncbi.nlm.nih.gov/35960887/), which reviewed 87 studies and found, "Compared with the patient-mediated approach, direct relative contact resulted in significantly higher uptake of genetic counseling for all relatives (63% [95% CI, 49 to 75] v 35% [95% CI, 24 to 48]) and genetic testing for first-degree relatives (62% [95% CI, 49 to 73] v 40% [95% CI, 32 to 48])."?	Thank you for this comment. We have clarified that previous studies on direct contact mainly are based on observational studies and no longer claim that we (completely) lack solid data from randomised studies. Please see page 10, line 19-21: *"On the other hand, it should be recognized that there is limited data from randomised studies on the effectiveness of HCP-led direct contact".*
**Page 10/34**	
Line 33: For a more recent analysis of the Australian position, see https://pubmed.ncbi.nlm.nih.gov/32805446/	We have referenced this analysis.

Line 39/40: I think you mean active NONdisclosure.	We have corrected to "nondisclosure".
**Page 12/24**	
Line 15-16: An important factor you have not noted is the trend in younger people towards viewing HCPs as having this obligation. Given you had an older population, and this trend still emerged, that is strong evidence that younger generations have a strong view. This may play out in your re-issue of the survey, but is worth briefly noting in this manuscript as well.	Thanks! We have now noted in the discussion that younger people are more prone to ascribe responsibility to HCPs, and we refer back to this observation in the methodological considerations. Please see page 11, line 33-37: *"The fact that younger people were more prone to ascribe moral responsibility to HCPs may indicate a generational shift".*<br><br>And page 12, line 53-56: *"The data was collected a few years ago, and there is a possibility of a shift in attitudes since then, especially since younger respondents are more prone to ascribe responsibility to HCPs."*
Reviewer: 2<br>Dr. Sandra Daack-Hirsch, University of Iowa College of Nursing	
Comments to the Author:<br>I thought the article was well written and straightforward. I think there is room for improvement by adding a bit more description and clarity to the following:	Thank you!
1. Page 5. Please include any laws or legislation that govern HCP responsibility for disclosing genetic results to ARR here rather than in the discussion. Are there ramifications for the HCP if they do disclose results to ARR?	We have added a paragraph about the legal situation in Sweden under the heading Context: Swedish Healthcare. Please see page 6, line 31-38: *"The Swedish national legislation does not address genetic counselling.[17] However, in the preparatory works to the Genetic Integrity Act (2006:351), it is noted that HCPs may inform ARRs directly about the results of a genetic test if the patient consents. Circumstances in each case should guide whether the disclosure to ARRs should be handled by the patient or by HCPs."*

Please describe how participants were randomly selected. What is the online research infrastructure?	We have now clarified the section about The citizen panel by describing how the sample initially were recruited and adding reference to the Citizen web page (in English). Please see Page 6, line 51-58;<br>*"Participants were recruited through the digital research infrastructure Citizen Panel, hosted by the Laboratory of Opinion Research (LORE) at the University of Gothenburg, Sweden.[20] We invited a stratified sample of panelists that had previously been recruited to the Citizen Panel from a randomly selected sample of the Swedish Population Register Survey data by distributing an electronic questionnaire.[21]".*
It seems to me that there could be correlations among findings from the various scenarios and the 5th scenario reported here. I wonder if the discussion might be more robust if the authors included a discussion that considered the findings from the other the scenarios as well.	Thank you for this helpful comment. We have now added selected data regarding participants preferences of risk information for themselves and their relatives – and performed subgroup analyses of the responses in relation to the data from this study (see supplement table 2, 3, 4, and 5). This new data is reflected in the Results (page 8, line 32-39:<br>*"Among those who would like to be informed about a potential risk for colorectal cancer, and those who wanted their relatives to be informed about such risk, a significantly larger proportion ascribed a moral responsibility to the patient, as well as to HCPs, compared to those who did not want to be informed, or did not want their relatives to be informed. (Supplementary Table 2)."*<br><br>and page 9, line 4-10:<br>*"Among those who would like to be informed about a potential risk for colorectal cancer, and those who wanted their relatives to be informed about such risk, a significantly larger proportion ascribed a legal responsibility to the patient, as well as HCPs, compared to those who did not*

	want to be informed, or did not want their relatives to be informed. (Supplementary Table 4).”<br><br>and page 9, line 20-27:<br>*Among those who would like to be informed about a potential risk for colorectal cancer, and those who wanted their relatives to be informed about such risk, a significantly larger proportion thought that HCP should inform ARRs against the patients will, as compared to those who did not want to be informed, or did not want their relatives to be informed (P<0.001).*<br><br>and in the Discussion, on page 11, line 36-46:<br>*“The only subgroups that diverge quite substantially from the majority are those who did not themselves want to be informed, and those who did not want their relatives to be informed. These subgroups are much less prone to ascribe moral responsibility, especially to the patient. This is unsurprising – if one does not want to be informed or one’s relatives to be informed, it makes sense to reject the idea that anyone should be responsible for informing.”*
I think the discussion was a somewhat redundant of the findings.	We have reduced the repetition of results in the discussion by cutting down several paragraphs.
**Minor typos:**	
Page 7. A value is missing in the first paragraph, line 7.	Corrected
Page 8. there are two commas, line 35.	Corrected
